# Chemerin as a Mediator of Hypertension and Cardiometabolic Diseases (A Comprehensive Review)

**DOI:** 10.1007/s11906-025-01354-3

**Published:** 2025-12-29

**Authors:** Ronald McMillan, Annet Kirabo

**Affiliations:** 1 Division of Infectious Diseases, Vanderbilt University Medical Center, Nashville, TN USA; 2 Division of Genetic Medicine and Clinical Pharmacology, Vanderbilt University Medical Center, Nashville, TN USA; 3 Vanderbilt Center for Immunobiology, Nashville, TN USA; 4 Vanderbilt Institute for Infection, Immunology, and Inflammation, Nashville, TN USA; 5 Vanderbilt Institute for Global Health, Nashville, TN USA; 6 Department of Molecular Physiology and Biophysics, Vanderbilt University, Nashville, TN USA

## Abstract

**Purpose:**

Chemerin, a recently identified adipokine, has emerged as a critical mediator in multiple physiological systems with significant implications for human health and disease. This review synthesizes current knowledge on chemerin’s multifaceted roles across cardiovascular, renal, immune, and metabolic processes. Acting through its primary receptor CMKLR1, chemerin influences sympathetic nervous system activity in key brain regions including the nucleus tractus solitarius and paraventricular nucleus, thereby regulating blood pressure and cardiovascular function.

**Recent Findings:**

In the kidneys, elevated chemerin levels correlate with declining renal function, serving as both a biomarker and pathogenic factor in chronic kidney disease progression and diabetic nephropathy. As an immune modulator, chemerin facilitates leukocyte recruitment, promotes macrophage polarization toward pro-inflammatory phenotypes, and enhances endothelial inflammation, establishing it as a pivotal link between metabolic dysregulation and chronic inflammatory states. Chemerin plays a central role in hypertension by altering endothelial function, renal function and sympathetic outflow. In metabolic regulation, chemerin influences adipocyte differentiation, glucose homeostasis, and central appetite control, connecting obesity with systemic inflammation and insulin resistance.

**Summary:**

The convergence of these diverse functions positions chemerin as an integrative signaling molecule with considerable therapeutic potential. This review highlights chemerin’s role as a promising target for novel interventions in hypertension, kidney disease, inflammatory disorders, and metabolic syndrome, potentially transforming treatment strategies for these interconnected conditions.

## Introduction

Cardiometabolic disease is influenced by unhealthy lifestyle factors that contribute to obesity, hypertension, and insulin resistance [1]. A staggering 40% of Americans are classified as obese [2]. The Western diet has significantly contributed to the global epidemic of obesity, which in turn increases the risk of developing serious health conditions such as type 2 diabetes, cardiovascular disease, and hypertension [3]. Excess adipose tissue has been linked to the development of the salt sensitive blood pressure (SSBP) phenotype, which is characterized by an elevated blood pressure in response to increased dietary sodium intake [4]. SSBP is associated with the development of hypertension [5] and a major risk factor for death and heart disease [6]. New evidence suggests that adipokines, which are cytokines derived from adipose tissue, may be a key factor in the mechanisms underlying SSBP [7–9], however these mechanisms have yet to be fully understood. Hence there is a gap in the field in regard to understanding the relationship between adipose, salt sensitivity, and vascular function.

Excess accumulation of visceral adipose tissue is a significant risk factor for the development of insulin resistance and hypertension [10]. The dysregulation of adipokines contribute to the pathogenesis of insulin resistance and hypertension. Chemerin is a novel adipokine that could serve as a potential link between obesity and the associated metabolic and cardiovascular complications [11]. In a study led by Cheon and colleagues, a cohort of 102 subjects newly diagnosed with type 2 diabetes had serum chemerin levels that strongly correlated with obesity and insulin resistance [12]. Further studies investigating the role of chemerin in skeletal tissue found that chemerin impaired insulin signaling, further contributing to the development of insulin resistance [13]. In a clinical trial led by Zang and colleagues, their group found that increased plasma chemerin levels were associated with increased BP [14]. In rodent studies conducted by Watts et al., they demonstrate that chemerin induces vasoconstrictive actions, connecting adipose tissue to arterial contraction [15]. This impact that adipose tissue has on vasculature tissues has been underexplored.

**Fig. 1:**
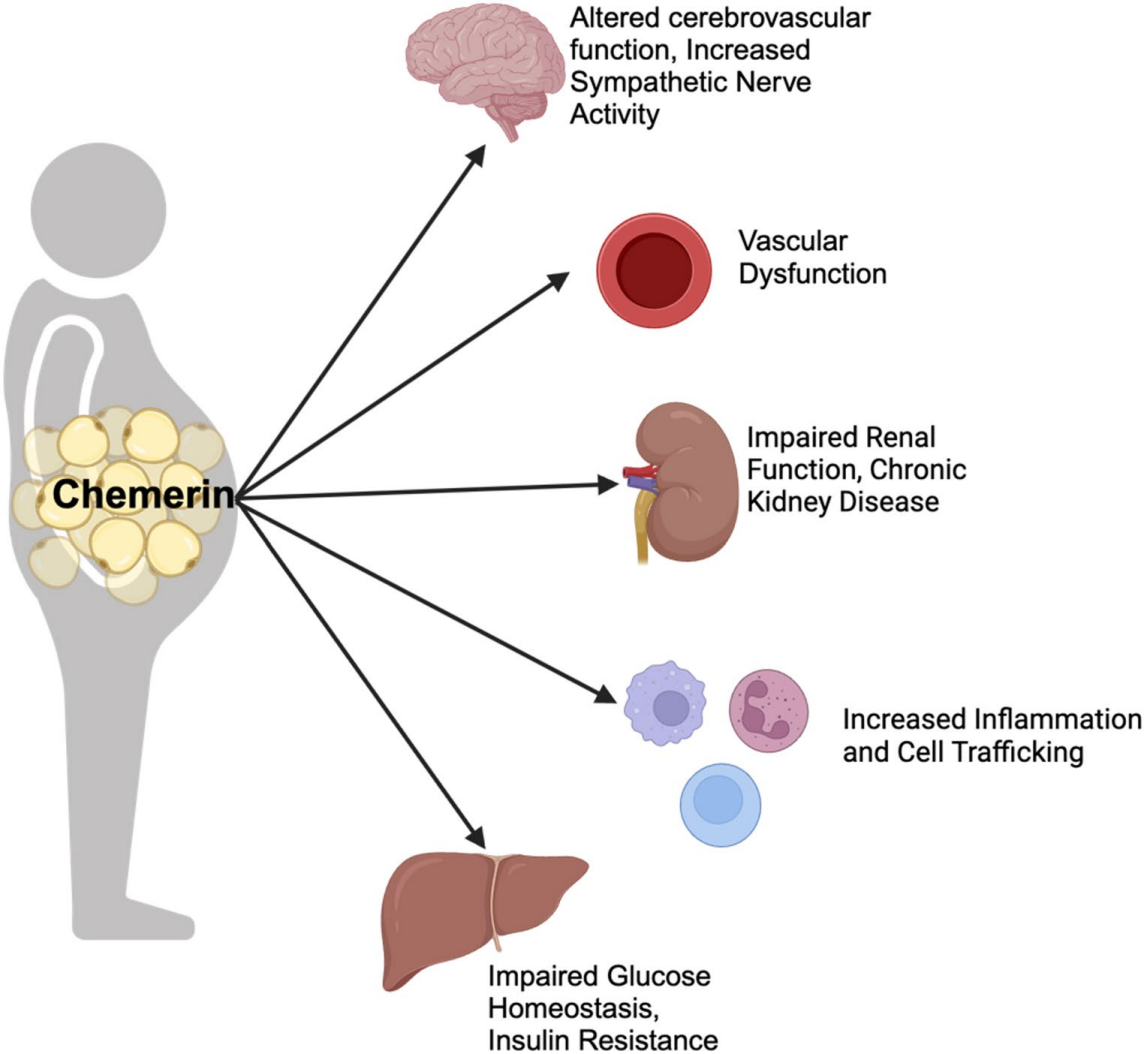
Effects of Chemerin on BP related tissues

Perivascular adipose tissue (PVAT) surrounds large arteries and organ-specific fat depots, like epicardial, pericardial, and peri-renal fat, that surround the blood vessels of organs [16]. Excess fat tissue increases the risk of blood pressure-related diseases by altering vascular structure, impairing kidney function, and increasing systemic inflammation. Identifying and targeting adipokines like chemerin could open up new therapeutic avenues for effectively managing hypertension in individuals with excess adipose tissue. As depicted in Fig. 1, this review aims to highlight relevant research that connects chemerin secretion to hypertension and insulin resistance while exploring its potential as a mediator in these processes.

### Chemerin Structure and Function

The first known adipokine to be discovered was leptin in 1994. Since then, more than 600 adipokines have been identified with chemerin being a novel adipokine with a discovery date in the year 2003 [17]. Chemerin levels were identified in human inflammatory fluids, with increased recruitment of antigen-presenting cells observed in response to chemerin signaling, supporting its classification as a chemotactic adipokine [18]. Chemerin is encoded by the retinoic acid receptor responder 2 (RARRES2) gene and regulates a variety of physiological processes, including inflammation, insulin resistance, and cardiovascular function. RARRES2, expressed in 27 human tissues including the adrenal gland, adipose tissue, and liver encodes a 163-amino acid protein. Unlike classical adipokines chemerin is unique in that it is secreted as a precursor protein, pro-chemerin, and requires further processing [19, 20]. Chemerin secretion also differs from leptin in that it is not constitutively released in proportion to adipocyte mass [21–23]. Instead, its expression is upregulated during adipocyte differentiation and expansion, with pre-adipocytes expressing low levels and mature adipocytes showing markedly higher RARRES2 expression [24–26]. This pattern suggests that chemerin coordinates immune–metabolic signaling during adipose tissue remodeling, whereas leptin secretion more directly reflects adipocyte size and energy storage. This precursor undergoes extracellular cleavage by serine proteases to produce the fully active 143-amino acid chemerin [18]. This sequence undergoes extracellular cleavage by serine proteases to produce the fully active 143-amino acid chemerin. Chemerin binds to three receptors, CMKLR1, GPR1, and CCRL2, with CMKLR1 serving as its primary receptor. Upon binding, CMKLR1 activates intracellular signaling pathways such as MAPK (mitogen-activated protein kinase) and AKT (protein kinase B), which regulate cellular processes including lipid metabolism, glucose uptake, and inflammation. Simultaneously, chemerin binding inhibits cAMP production, potentially affecting lipolysis and energy homeostasis.

Adipocytes secrete chemerin in response to increased BMI and adipocyte volume, as demonstrated by Sell and colleagues, who observed a direct correlation between chemerin secretion and adipose tissue expansion [13]. This suggests a role for chemerin as a sensor of adipose tissue expansion, linking adipose tissue growth to systemic metabolic signaling. Interestingly, their findings revealed sex-specific differences in chemerin expression, suggesting hormonal or genetic factors may modulate its levels [13]. Both chemerin and its primary receptor, CMKLR1, are highly expressed in adipocytes, playing a pivotal role in the differentiation and regulation of genes involved in lipid and glucose metabolism [26]. Secreted chemerin mediates the recruitment of CMKLR1-expressing cells to adipose tissue, triggering intracellular signaling pathways, including the activation of ERK1/2 signaling [27]. This signaling provides strong evidence of autocrine and paracrine functions facilitated by CMKLR1 activation [26].

The regulation of chemerin is influenced by multiple factors, including environmental, hormonal, and epigenetic mechanisms. High-fat diets and sedentary behavior have been linked to elevated chemerin levels, which exacerbate inflammation and metabolic dysfunction [28]. Hormones such as cortisol, insulin, and sex steroids [29] further modulate its expression, while epigenetic modifications, such as methylation of the RARRES2 promoter, suggest a gene-environment interaction. Experimental and clinical studies have highlighted the physiological and pathological significance of chemerin [30]. Elevated plasma chemerin levels have been observed in patients with obesity, type 2 diabetes, and cardiovascular diseases, and these levels often decrease following weight-loss interventions like bariatric surgery or calorie restriction [31]. In animal models, chemerin overexpression has been associated with increased adiposity, impaired glucose tolerance [32], and chronic inflammation, while chemerin deletion has shown protective effects [33]. In vitro experiments reveal that chemerin influences macrophage polarization, promoting a pro-inflammatory phenotype in adipose tissue [34].

Chemerin and CMKLR1 represent promising therapeutic targets for treating metabolic and inflammatory diseases. The development of CMKLR1 antagonists or neutralizing antibodies could mitigate the pro-inflammatory and insulin-resistant effects of chemerin, while interventions targeting the extracellular cleavage of pro-chemerin may reduce its pathological activity. Additionally, chemerin’s potential as a biomarker for metabolic disorders could aid in disease diagnosis and therapeutic monitoring. Future research should further elucidate the roles of chemerin’s additional receptors, GPR1 and CCRL2, to fully understand its signaling network. These advancements may unlock new therapeutic strategies, making chemerin a critical focus in addressing metabolic and inflammatory diseases.

### Impact of Chemerin on BP Regulation

#### Endothelial Function

Chemerin modulates endothelial function by maintaining vascular homeostasis and regulating vascular tone [35]. This is done primarily through its impact on inflammatory processes and vascular remodeling. The endothelium serves as the interface between circulating blood and vessel walls and requires a delicate balance to function properly [36]. Chemerin appears to both support and disrupt mechanisms that contribute to this balance depending on context and concentration. Research has revealed several key mechanisms through which chemerin affects vascular health. In a study led by Wabel et al. used rodent models and found that chemerin was detected immunohistochemically in the endothelium, smooth muscle cells, adventitia, and perivascular adipose tissue [37]. Furthermore, chemerin colocalized with the vascular smooth muscle marker α-actin. Xiong and colleagues demonstrated in their in vitro work that inhibiting chemerin prevented vascular smooth muscle cell proliferation while simultaneously reducing pro-inflammatory cytokine activation [38]. These findings highlight chemerin’s significant role in vascular inflammation and suggest potential therapeutic applications in conditions characterized by excessive vascular smooth muscle cell growth, such as restenosis following angioplasty or atherosclerotic plaque formation.

Rodent studies have further illuminated chemerin’s impact on blood vessels, providing crucial in vivo evidence of its physiological effects [39]. It has been linked to structural blood vessel remodeling and elevated systolic blood pressure in mice, suggesting a direct role in hypertension pathophysiology [40]. In a particularly revealing study, Neves and colleagues found that blocking the chemerin receptor CMKLR1 in diabetic obese mice enhanced vascular insulin signaling through redox-sensitive and Akt-dependent pathways [41]. This finding bridges the gap between metabolic disorders and vascular complications, suggesting chemerin may represent a molecular link between obesity, diabetes, and cardiovascular disease.

Human studies complement these findings, providing clinical relevance to chemerin’s vascular effects. Gu and colleagues observed that hypertensive patients with elevated chemerin levels showed impaired endothelial function and increased arterial stiffness, as measured by flow-mediated dilation [42]. This clinical correlation strengthens the case for chemerin’s involvement in human vascular pathology and suggests its potential utility as a biomarker for endothelial dysfunction. Perhaps most compelling, Spiroglou and colleagues discovered that higher chemerin concentrations in periaortic and pericoronary fat correlated with aortic and coronary atherosclerosis in 41 autopsy cases, suggesting localized effects on atherosclerosis development [43]. This observation points to the importance of adipose tissue-derived chemerin in creating microenvironments conducive to atherosclerotic lesion formation, a concept that advances our understanding of how adipokines contribute to cardiovascular disease.

What makes chemerin particularly intriguing is its apparent contradictory effects on vascular function, revealing a biological duality that challenges simplistic categorization. On one hand, it promotes inflammation by activating the NF-κB pathway, increasing adhesion molecule expression, and enhancing monocyte-endothelial adhesion [44], all processes that contribute to endothelial dysfunction and early atherosclerosis. On the other hand, it demonstrates anti-inflammatory properties by activating the Akt/eNOS pathway, which boosts nitric oxide production and inhibits TNF-α-induced VCAM-1 expression and monocyte adhesion [45]. This paradoxical nature suggests that chemerin’s net effect might depend on the specific microenvironment, receptor availability, and concurrent signaling pathways active in the vasculature at any given time.

In vascular smooth muscle cells, chemerin exerts equally complex effects that collectively influence vessel tone and structure. It stimulates proliferation and migration through ROS-dependent phosphorylation of Akt and ERK signaling pathways, processes central to vascular remodeling in both physiological adaptation and pathological states [46]. Chemerin induces vasoconstriction by interacting with receptors in smooth muscle cells, which reduces cAMP levels and increases ERK1/2 and ROS signaling, contributing to acute changes in vascular tone and blood pressure regulation [47]. Additionally, chemerin indirectly influences vasoconstriction through sympathetic nervous system activation, highlighting its integration with broader physiological control systems beyond direct cellular effects.

Together, these mechanisms reveal how chemerin functions as a multifaceted regulator that modulates vascular tone, drives structural remodeling, and contributes to endothelial dysfunction, highlighting its significance in cardiovascular health and disease progression. The intricate network of chemerin’s actions represents a promising target for therapeutic intervention, potentially offering new approaches to address the growing burden of cardiovascular diseases. Further research into the contextual factors that determine whether chemerin promotes vascular health or disease could yield valuable insights for personalized medicine approaches to cardiovascular conditions.

#### Sympathetic Nervous Activity

Chemerin exerts significant influence on sympathetic nervous activity through critical brain regions such as the nucleus tractus solitarius (NTS) and paraventricular nucleus (PVN). Research shows that chemerin-9, an active fragment of chemerin, enhances renal sympathetic nerve activity (RSNA), mean arterial pressure (MAP), and heart rate (HR) by activating the chemokine-like receptor 1 (CMKLR1) pathway [48]. Hao and colleagues demonstrated that chemerin-9 injection into the NTS of rats increased both RSNA and MAP, suggesting this activation pathway regulates sympathetic outflow and central blood pressure [49]. This process increases oxidative stress and superoxide production through NADPH oxidase activation in the NTS, which connects directly to sympathetic nerve activity and blood pressure regulation. Further research revealed that when administered into the PVN, chemerin-9 increases RSNA and MAP through glutamate receptor-mediated calcium influx and reactive oxygen species (ROS) generation [48]. This finding reinforces the central role of chemerin in sympathetic activation. Moreover, chemerin stimulates catecholamine release from the adrenal medulla, amplifying sympathetic activity and contributing to systemic blood pressure control [50]. Su and colleagues observed elevated catecholamine levels following chemerin administration, further supporting its involvement in enhancing sympathetic responses.

The regulatory role of chemerin extends to perivascular adipose tissue (PVAT), where it co-localizes with sympathetic nerve markers, indicating direct involvement in vascular tone modulation. Ray and team found abundant chemerin expression in PVAT surrounding the aorta. Activation of CMKLR1 in adipocytes increases inflammatory cytokine and ROS release, contributing to vascular remodeling and tone regulation [50]. This suggests chemerin influences not only the sympathetic nervous system but also local vascular responses, potentially impacting hypertension development. Blood pressure studies demonstrate that CMKLR1 activation in the PVN and subfornical organ raises systemic blood pressure [49]. Neves and colleagues showed that CMKLR1 blockade in diabetic obese mice reduced blood pressure response to chemerin administration, indicating chemerin affects blood pressure through sympathetic activation and the CMKLR1 receptor [41]. Similarly, CMKLR1 silencing in Wistar rats significantly reduced blood pressure following chemerin injection, confirming chemerin’s role in blood pressure regulation through sympathetic activation [51]. In these models, CMKLR1 silencing alleviated the hypertensive effects of chemerin, establishing a direct link between chemerin-induced sympathetic activity and blood pressure regulation.

While sympathetic activity clearly influences blood pressure and kidney function, the specific effects of chemerin-induced sympathetic nerve activation in adipose tissue on renal function and blood pressure regulation require further investigation. Madsen and colleagues suggest chemerin’s effects in adipose tissue might involve local sympathetic activation influencing kidney function and fluid retention, though these mechanisms need clarification through additional research. Multiple studies support chemerin’s dual role in promoting vasoconstriction and regulating systemic blood pressure. Spiroglou and team linked elevated chemerin levels in periaortic and pericoronary fat to increased aortic and coronary atherosclerosis in autopsy cases [43]. This localized effect on vascular structures indicates chemerin’s actions extend beyond central nervous system regulation to direct modulation of vascular function and remodeling. The complexity of chemerin’s interactions with various receptors, primarily CMKLR1, underscores the need for further exploration of its role in sympathetic regulation and cardiovascular health.

Future research should investigate how fluctuations in chemerin expression affect sympathetic nerve-mediated responses across various organs, particularly the kidney and vasculature. Understanding the mechanisms through which chemerin regulates blood pressure and sympathetic activity could provide valuable insights for developing targeted therapies for hypertension and cardiovascular diseases linked to dysregulated sympathetic responses.

#### Renal Function

Research increasingly implicates chemerin, an adipokine, in renal dysfunction, particularly in chronic kidney disease (CKD) and diabetes. Elevated serum chemerin levels correlate with impaired renal function, demonstrated by a negative relationship with estimated glomerular filtration rate (eGFR) [52, 53]. In a systemic review, CKD patients exhibit significantly higher chemerin levels compared to healthy controls, with these elevated levels strongly correlating with declining eGFR [54]. This association worsens with metabolic syndrome, which accelerates kidney damage through inflammation, oxidative stress, and lipid metabolism dysregulation. Both CKD and diabetes patients with higher chemerin levels experience worse renal outcomes. Longitudinal data reveals that each 25 ng/ml increase in serum chemerin links to a threefold higher risk of developing CKD, highlighting chemerin’s potential as a predictive biomarker [55]. In diabetic patients, chemerin levels correlate with key kidney function parameters, including albuminuria and creatinine levels, making it valuable for early detection of renal dysfunction [56]. Chemerin contributes to renal fibrosis, a hallmark of CKD, through fibroblast activation and extracellular matrix deposition [57]. Wang and colleagues demonstrated that chemerin induces fibroblast proliferation and promotes collagen synthesis, supporting its role in renal fibrosis progression [58].

The interaction between chemerin and endothelial cells affects vascular integrity and kidney function. In vitro studies show that chemerin disrupts endothelial cell barrier function, increasing vascular permeability and worsening kidney injury [52]. Animal models of diabetes provide evidence that blocking chemerin receptors, particularly CMKLR1, offers renoprotective effects. Research by Neves and team demonstrated that CMKLR1 antagonism reduced oxidative stress, improved renal blood flow, and enhanced kidney function in diabetic rats [41]. These findings highlight the therapeutic potential of targeting chemerin pathways to mitigate renal damage. Chemerin levels normalize following kidney transplantation [59], further supporting its utility as a renal function marker. Studies on chronic hemodialysis patients show that elevated serum chemerin levels independently associate with renal dysfunction markers, including urea and creatinine, demonstrating its diagnostic value in advanced kidney disease [60]. However, some research suggests elevated chemerin levels in CKD may partly result from impaired renal clearance rather than increased production [54].

Chemerin also shows associations with lipid metabolism, as its levels correlate with serum cholesterol, LDL, and triglycerides [61], suggesting dietary factors may influence its production. This connection to lipid profiles further links chemerin to metabolic syndrome pathogenesis and its impact on renal health. These findings suggest managing dietary and metabolic factors could help modulate chemerin levels and improve renal function. Despite significant advances in understanding chemerin’s role in renal dysfunction, many questions remain unanswered. Future research should clarify the mechanisms by which chemerin contributes to kidney injury, including its potential involvement in inflammatory signaling, oxidative stress pathways, and crosstalk between adipose tissue and renal cells. Additionally, clinical studies evaluating chemerin-targeted therapies could pave the way for novel treatments for CKD and diabetes-associated kidney damage.

#### Cardiac Tissue

Emerging evidence suggests that chemerin is active in epicardial adipose tissue (EAT), where it may directly affect cardiac function and local inflammation [62–64]. Elevated chemerin expression in EAT correlates with epicardial fat thickness and cardiovascular risk. In patients with rheumatoid arthritis, Ekinci et al. reported that chemerin levels positively associated with epicardial fat tissue, while low adropin levels were linked to increased cardiovascular involvement [62]. Similarly, studies in morbid obesity found that chemerin and the adiponectin/chemerin ratio correlated with carotid intima-media thickness and arterial stiffness, suggesting its value as an early marker of cardiovascular dysfunction [65]. Experimental models also support a direct role for chemerin in the heart. In an ex vivo rat model, acute chemerin treatment induced a negative inotropic effect mediated through nitric oxide, voltage gated channel Ca_V_1.2, and PI3Kγ pathways, demonstrating its capacity to alter myocardial contractility [64]. Chemerin promotes inflammation, oxidative stress, and lipid imbalance in the heart, contributing to atherosclerosis, fibrosis, and arrhythmias [66–68]. Together, these findings suggest that chemerin contributes to EAT–heart crosstalk in a manner similar to its established effects in the kidney, where it drives tissue-specific inflammation and remodeling. By highlighting chemerin’s organ-specific roles, particularly in EAT, this perspective strengthens the rationale for targeting chemerin pathways to mitigate cardiovascular disease risk.

#### Inflammatory Response

Chemerin serves as a pivotal regulator of immune system function, particularly in inflammation [69] and immune cell trafficking [70]. Elevated chemerin levels strongly correlate with heightened inflammatory responses in autoimmune conditions such as rheumatoid arthritis [71] and systemic lupus erythematosus [72], suggesting its role in perpetuating chronic inflammation. Functioning as a chemoattractant, chemerin facilitates the migration of innate immune cells, including macrophages and dendritic cells, to sites of injury or inflammation [73]. This process occurs primarily through its interaction with CMKLR1, a receptor expressed on various immune cells [74]. When binding to CMKLR1, chemerin modulates leukocyte recruitment by inhibiting cAMP and activating PI3K/Akt and ERK1/2 signaling pathways, which play critical roles in immune cell migration and activation [75]. Additionally, chemerin interacts with CCRL2, a receptor that enhances its local concentration at inflammation sites [74]. This mechanism amplifies chemerin’s effects in localized immune responses. In macrophages, chemerin induced phenotypes are mediated by Akt activation and the transcription factor CEBPα [76]. CEBPα functions as a transcriptional activator for chemerin itself and IRF8, a key regulator of dendritic cell lineage specification and monocyte/macrophage development [76, 77]. Through these pathways, chemerin promotes the polarization of macrophages toward the pro inflammatory M1 phenotype, exacerbating tissue damage and disease progression in chronic inflammatory conditions.

The pro inflammatory effects of chemerin extend to its role in macrophage adhesion and migration [78]. It enhances macrophage adhesion to extracellular matrix proteins and adhesion molecules through integrin clustering, contributing to inflammation persistence. Hart and Greaves noted that chemerin’s impact on macrophage behavior significantly influences the inflammatory microenvironment. Similarly, chemerin induces endothelial cell inflammation by activating NF κB signaling, which upregulates the expression of adhesion molecules including E selectin, VCAM 1, and ICAM 1 [78]. These molecules facilitate monocyte endothelial adhesion, a critical step in recruiting immune cells to inflamed tissues, as demonstrated by Dimitriadis and colleagues [44]. Beyond autoimmune diseases, chemerin plays significant roles in metabolic and reproductive health disorders [23, 79]. Patients with polycystic ovary syndrome (PCOS) exhibit significantly elevated serum chemerin levels and increased chemerin expression in ovarian tissue [80], linking it to inflammatory and metabolic disturbances characteristic of this condition. In obese individuals, elevated chemerin levels positively correlate with markers of metabolic syndrome, including dyslipidemia and insulin resistance [81]. Lehrke and colleagues demonstrated that chemerin strongly associates with inflammatory biomarkers such as C reactive protein, interleukin 6, and tumor necrosis factor alpha [82], further underscoring its role in systemic inflammation.

Interestingly, while chemerin associates with inflammation and metabolic syndrome, its direct relationship with coronary atherosclerosis remains inconclusive [83, 84]. Lehrke and team found that after adjusting for established cardiovascular risk factors, the association between chemerin levels and coronary atherosclerosis was no longer significant [82]. This suggests chemerin’s role in cardiovascular disease may be mediated through its effects on systemic inflammation and metabolic regulation rather than direct vascular pathology. The multifaceted immune modulatory effects of chemerin highlight its therapeutic potential in conditions characterized by dysregulated inflammation and immune activation [85]. Targeting chemerin or its receptors could provide novel strategies for treating autoimmune diseases, chronic inflammatory conditions, and metabolic disorders. However, further research must clarify chemerin’s precise mechanisms of action and its interplay with other immune and metabolic pathways. Understanding these mechanisms will prove crucial for developing effective interventions to modulate chemerin activity and alleviate disease progression.

#### Chemerin Contributes to Insulin Resistance

Chemerin disrupts insulin signaling by modulating adipogenesis, impairing glucose homeostasis, and promoting inflammation. Secreted primarily by adipocytes, chemerin levels are significantly elevated in obese individuals and show a strong positive correlation with body mass index (BMI) and markers of metabolic syndrome, including insulin resistance, dyslipidemia, and hypertension [26]. In adipose tissue, chemerin serves as a critical regulator of adipocyte differentiation and metabolism [13, 26], while its release is associated with impaired lipogenesis and reduced insulin-induced anti-lipolysis, further linking it to insulin resistance [86]. In skeletal muscle cells, chemerin hinders glucose uptake by impairing insulin signaling pathways), reinforcing its role in systemic metabolic dysfunction [13].

Emerging evidence underscores the diverse effects of the chemerin/CMKLR1 axis across metabolic tissues. In women with polycystic ovary syndrome (PCOS), elevated chemerin expression in granulosa-lutein cells is associated with reduced insulin sensitivity due to impaired IRS1/2 and Akt phosphorylation [87]. Antagonism of CMKLR1 using α-NETA, alone or combined with adipose-derived stem cells, improves metabolic and endocrine parameters in a PCOS rat model, highlighting a potential therapeutic approach [88]. In the context of pancreatogenic diabetes, reduced chemerin levels correlate with increased insulin resistance, while treatment with the CMKLR1 agonist chemerin-9 improves glucose tolerance and restores GLUT2 and PDX1 expression, suggesting a protective role for chemerin signaling [89]. Similarly, in models of nonalcoholic steatohepatitis (NASH), chemerin improves insulin sensitivity by reducing oxidative stress and activating autophagy via the JAK2-STAT3 pathway—effects reversed by CMKLR1 inhibition [90]. In contrast, PI3K inhibition downregulates CMKLR1 and NLRP3 in Kupffer cells, protecting against hepatic insulin resistance and inflammation in diet-induced liver disease, indicating that CMKLR1’s role may vary by context [91]. Chemerin also contributes to obesity-related metaflammation. In adipose tissue, it recruits plasmacytoid dendritic cells (pDCs) via CMKLR1, initiating a type I interferon response that promotes proinflammatory macrophage activation and correlates with both tissue and systemic insulin resistance [92]. Beyond inflammation, chemerin impacts energy metabolism through its regulation of thermogenesis. CMKLR1 deficiency exacerbates glucose intolerance and impairs thermogenic gene expression in mice on a high-fat diet [93]. Moreover, chemerin and resolvin E1, which both signal through CMKLR1, exert opposing effects on beige fat development: chemerin suppresses thermogenesis and worsens obesity, while resolvin E1 enhances thermogenic activity and metabolic health via mTORC1 signaling [94].

Chemerin also modulates central appetite regulation and energy balance. Elevated chemerin promotes hyperphagia and weight gain, while its inhibition improves metabolic outcomes [26, 81]. In rodents, although chemerin did not directly alter feeding behavior, it increased hypothalamic expression of appetite-related genes, including Agouti-related protein (AgRP) and Proopiomelanocortin (POMC) [95]. CMKLR1 is also expressed on tanycytes, specialized glial cells lining the third ventricle of the hypothalamus, which are closely linked to appetite regulation [96]. Unlike leptin, which suppresses appetite and reduces body weight, chemerin appears to have the opposite effect on food intake and weight gain, underscoring its distinct role in hypothalamic circuits [97, 98]. This paradox is particularly notable given that both chemerin and leptin enhance sympathetic nervous system activity, suggesting possible convergence or divergence in their central signaling mechanisms [49, 99]. Chemerin has been shown to modulate hypothalamic feeding circuits, including effects on AgRP/NPY neurons in the arcuate nucleus, which are key drivers of food intake, with potential downstream influence on regions such as the paraventricular nucleus [100]. Together, these findings highlight chemerin’s unique influence on hypothalamic control of energy homeostasis and raise important questions about how its signaling intersects with, or diverges from, leptin pathways. Further investigation of these mechanisms could provide new insights into the neuroendocrine regulation of metabolism.

Building on these insights into chemerin’s central actions, therapeutic strategies that target chemerin signaling may also hold metabolic benefits. For example, weight loss through caloric restriction or bariatric surgery reduces chemerin levels, improving insulin sensitivity and inflammatory profiles [86]. Experimental CMKLR1 antagonists have also shown promise in reversing insulin resistance and reducing adipose inflammation [33]. Together, these findings position chemerin as a critical mediator linking obesity, inflammation, and metabolic dysfunction. Its context-specific actions across adipose, hepatic, ovarian, and central tissues highlight the need for targeted strategies to modulate chemerin signaling in metabolic disease.

## Conclusion

Chemerin emerges as a multifunctional adipokine with profound implications across numerous physiological systems. Its diverse roles span sympathetic nervous system regulation, renal function, immune modulation, blood pressure control, and metabolic regulation. Through its interactions with receptors such as CMKLR1, chemerin influences sympathetic outflow, vascular tone, and blood pressure, particularly in salt-sensitive hypertension where it promotes sodium retention and systemic inflammation. In kidney function, chemerin serves as both a biomarker and mediator of renal dysfunction, with elevated levels correlating with declining eGFR and worse outcomes in CKD and diabetic nephropathy. Its immune modulatory effects, including macrophage recruitment, polarization toward pro-inflammatory phenotypes, and enhancement of endothelial inflammation, establish chemerin as a critical link between metabolic disorders and chronic inflammatory states. Furthermore, chemerin’s central role in adipogenesis, glucose homeostasis, and central regulation of food intake positions it as a key mediator in obesity-related metabolic dysfunction. Collectively, these multifaceted functions highlight chemerin as an integrative signaling molecule connecting adipose tissue biology, immune function, and cardiovascular health. The convergence of these pathways underscores chemerin’s potential as a therapeutic target for treating various conditions including hypertension, renal dysfunction, inflammatory disorders, and metabolic syndrome. Future research focusing on specific chemerin-mediated pathways could yield novel therapeutic strategies with applications across multiple disease states, potentially revolutionizing treatment approaches for these interconnected conditions.

## Data Availability

No datasets were generated or analysed during the current study.
